# Light induces NLRP3 inflammasome activation in retinal pigment epithelial cells via lipofuscin-mediated photooxidative damage

**DOI:** 10.1007/s00109-015-1275-1

**Published:** 2015-03-18

**Authors:** Carolina Brandstetter, Lena K. M. Mohr, Eicke Latz, Frank G. Holz, Tim U. Krohne

**Affiliations:** 1 Department of Ophthalmology, University of Bonn, Ernst-Abbe-Str. 2, 53127 Bonn, Germany; 2 Institute of Innate Immunity, University of Bonn, Bonn, Germany; 3 German Center for Neurodegenerative Diseases (DZNE), Bonn, Germany; 4 Department of Infectious Diseases and Immunology, University of Massachusetts Medical School, Worcester, MA USA; 5 Centre of Molecular Inflammation Research, Department of Cancer Research and Molecular Medicine, Norwegian University of Science and Technology (NTNU), Trondheim, Norway

**Keywords:** Age-related macular degeneration, Retinal pigment epithelium, Interleukin-1β, Lysosomal membrane permeabilization, Lipid peroxidation

## Abstract

**Abstract:**

Photooxidative damage and chronic innate immune activation have been implicated in retinal pigment epithelium (RPE) dysfunction, a process that underlies blinding diseases such as age-related macular degeneration (AMD). To identify a potential molecular link between these mechanisms, we investigated whether lipofuscin-mediated phototoxicity activates the NLRP3 inflammasome in RPE cells in vitro. We found that blue light irradiation (dominant wavelength 448 nm, irradiance 0.8 mW/cm^2^, duration 6 h) of lipofuscin-loaded primary human RPE cells and ARPE-19 cells induced photooxidative damage, lysosomal membrane permeabilization (79.5 % of cells vs. 3.8 % in nonirradiated controls), and cytosolic leakage of lysosomal enzymes. This resulted in activation of the inflammasome with activation of caspase-1 and secretion of interleukin-1β (14.6 vs. 0.9 pg/ml in nonirradiated controls) and interleukin-18 (87.7 vs. 0.2 pg/ml in nonirradiated controls). Interleukin secretion was dependent on the activity of NLRP3, caspase-1, and lysosomal proteases cathepsin B and L. These results demonstrate that accumulation of lipofuscin-like material in vitro renders RPE cells susceptible to phototoxic destabilization of lysosomes, resulting in NLRP3 inflammasome activation and secretion of inflammatory cytokines. This new mechanism of inflammasome activation links photooxidative damage and innate immune activation in RPE pathology and may provide novel targets for therapeutic intervention in retinal diseases such as AMD.

**Key message:**

• Visible light irradiation of lipofuscin-loaded RPE cells activates inflammasome.

• Inflammasome activation results from lysosomal permeabilization and enzyme leakage.

• Inflammasome activation induces secretion of inflammatory cytokines by RPE cells.

• Photooxidative damage by visible light as new mechanism of inflammasome activation.

• Novel link between hallmark pathogenetic features of retinal degenerative diseases.

## Introduction

Age-related macular degeneration (AMD) is a neurodegenerative disease of the retina that represents the most common cause of blindness in all industrialized countries [1]. The molecular pathogenesis of AMD is still not completely resolved which hinders the development of effective therapies, in particular for the atrophic subtype of the disease. In AMD, the retinal pigment epithelium (RPE) becomes progressively dysfunctional and eventually degenerates, resulting in photoreceptor death and visual function loss. Experimental and clinical studies identified oxidative and photooxidative damage to the RPE as a contributing factor [2, 3]. This damage is thought to be mediated, at least in part, by the phototoxic properties of lipofuscin that accumulate in RPE cells over a lifetime as a result of constant photoreceptor outer segment phagocytosis and degradation. Moreover, genetic and histochemical evidence supports a role of chronic innate immune activation at the level of the RPE in AMD pathology [4, 5]. A candidate innate immune signaling receptor for RPE cell pathology in AMD pathogenesis is the nucleotide-binding oligomerization domain-like receptor family, pyrin domain-containing protein 3 (NLRP3) inflammasome [6, 7].

Two steps are required for activation of the NLRP3 inflammasome [8]. First, a priming signal results in the transcriptional induction of NLRP3 and pro-interleukin (IL)-1β. Second, an activation signal triggers the assembly of NLRP3, apoptosis-associated speck-like protein containing a caspase recruitment domain (ASC), and pro-caspase-1 into the inflammasome protein complex. The activated inflammasome mediates caspase-1 activation, which in turn proteolytically activates pro-IL-1β and pro-IL-18 into their mature forms. These highly pro-inflammatory cytokines have pleiotropic autocrine and paracrine effects on a variety of cell types. NLRP3 inflammasome activation in the RPE has been reported in both atrophic and neovascular AMD [6, 7]. Several substances have been suggested to provide the signal for inflammasome activation in AMD including drusen components such as C1q [9] and amyloid-beta [10], Alu RNA accumulation secondary to DICER1 deficiency [6], the lipofuscin component *N*-retinylidene-*N*-retinyl-ethanolamine (A2E) [11], and the lipid peroxidation product 4-hydroxynonenal (HNE) [12].

A key pathway upstream of the NLRP3 inflammasome is lysosomal membrane permeabilization (LMP) and subsequent cytosolic leakage of lysosomal components [8, 13]. Indeed, this mechanism has been reported to activate the inflammasome in RPE cells following chemical induction of LMP [7]. Another mechanism resulting in LMP in RPE cells is phototoxic damage mediated by intralysosomal accumulation of photoreactive lipofuscin [14, 15]. In this study, we investigated whether blue light-induced photooxidative stress in human RPE cells, intensified by accumulated lipofuscin, and subsequent lysosomal membrane permeabilization result in activation of the NLRP3 inflammasome by leaking lysosomal enzymes. Thereby, this study aimed to identify a novel mechanism of inflammasome activation by light damage that may provide new treatment targets for blinding diseases such as AMD.

## Material and methods

### Cell culture and treatments

Human fetal primary RPE cells (pRPE) were obtained from Lonza (Cologne, Germany), cultured as recommended by the manufacturer, and used in experiments for a maximum of six passages. The human nontransformed RPE cell line ARPE-19 (CRL-2302; ATCC, Rockville, MD, USA) was cultured as previously reported [16]. The immortalized murine wild-type and NLRP3 knockout macrophage cell lines used in our study have been characterized previously [13] and were cultured as described [17].

For inhibition of photooxidative damage, the singlet oxygen scavenger 1,4-diazabicyclooctane (DABCO; Sigma-Aldrich, Munich, Germany) was added to the culture media at a concentration of 30 mM during blue light irradiation. Lysosomal membrane permeabilization was induced by incubation of cells with 1 mM ciprofloxacin (Sigma-Aldrich, Munich, Germany) for 3 h or 1 mM l-leucyl-l-leucine methyl ester (Leu-Leu-OMe; Bachem, Bubendorf, Switzerland) for 3 h. Cathepsin B inhibitor CA-074 (Calbiochem, Darmstadt, Germany) and cathepsin L inhibitor Z-Phe-Phe-fluoromethylketone (Z-FF-FMK; Calbiochem) were used for cell treatment at a concentration of 10 μM each for 1 h prior to and during irradiation treatment. For inhibition of caspase-1, we applied 10 μM of Z-Tyr-Val-Ala-Asp-fluoromethylketone (Z-YVAD-FMK; BioVision, Munich, Germany) 30 min prior to and during irradiation.

### Induction of lipofuscin accumulation

Isolated porcine POS were modified with lipid peroxidation products malondialdehyde (MDA) or 4-hydroxynonenal (HNE) as described [18]. Lipofuscin accumulation was induced by incubation of cells with modified POS for 7 days or, alternatively, by incubation with native POS and concomitant lysosomal inhibition by ammonium chloride (NH_4_Cl; Sigma-Aldrich, Munich, Germany) as reported previously [16]. Intracellular lipofuscin granules were documented by fluorescence microscopy (IX71; Olympus, Hamburg, Germany) using a fluorescein filter set (excitation filter wavelength 480/40 nm, emission filter wavelength 535/50 nm). For flow cytometric quantification (FACS Canto II; BD Biosciences, Heidelberg, Germany), we employed the FITC channel (excitation laser wavelength 488 nm, detection filter wavelength 530/30 nm). Data was acquired and analyzed by appropriate software (DIVA; BD Bioscience, Heidelberg, Germany; FlowJo; Tree Star, Ashland, OR, USA).

### Blue light irradiation

For blue light irradiation of RPE cells, we employed a 3 × 3 array of blue LEDs (XLamp XP-E royal blue; Cree, Durham, NC, USA). The LED spectrum as measured by a spectroradiometer (PR-655 SpectraScan; Photo Research, Chatsworth, CA, USA) exhibited a peak wavelength of 448 nm with a full width at half maximum (FWHM) of 24 nm. Cells were irradiated from a distance of 35 cm within a cell culture incubator for the indicated times. In this setup, cells were exposed to an irradiance of 0.8 mW/cm^2^ as measured using a power meter (FieldMax-TOP with PM10V1 sensor; Coherent, Santa Clara, CA, USA). Measurements confirmed that irradiation did not affect the temperature of the cell culture medium. A dose–response curve of the phototoxic effect of irradiation treatment on RPE cells is provided in Fig. 2b (control group). Photooxidative damage in blue light-irradiated cells was assessed by immunostaining of protein carbonyls using a commercially available kit (OxyICC Oxidized Protein Detection Kit; Merck Millipore, Darmstadt, Germany) according to manufacturer’s instructions, followed by quantification by flow cytometry (FACS Canto II; BD Bioscience, Heidelberg, Germany).

### Cell death detection assays

Blue light-induced cell death was documented by light microscopy. Cell death-associated plasma membrane disintegration was assessed by means of release of cytoplasmic lactate dehydrogenase (LDH). Measurements of LDH release were performed in cell supernatants 48 h after the start of irradiation using a calorimetric assay (Cytotoxicity Detection Kit; Roche, Mannheim, Germany) according to the manufacturer’s instructions. As an additional cell death assay, we quantified loss of cell attachment 48 h after the start of irradiation by crystal violet assay as previously described [19].

### Analysis of lysosomal membrane permeabilization

Cells were incubated with 1 μg/ml acridine orange (Sigma-Aldrich, Munich, Germany) for 15 min and washed two times with PBS. Lysosomal staining (red) was documented by fluorescence microscopy. Lysosomal permeabilization was quantified by flow cytometry (FACS Canto II; BD Bioscience, Heidelberg, Germany) as percentage of cells with loss of lysosomal staining. For analysis of leakage of lysosomeal enzymes, lysosomal and cytosolic cell fractions were separated following plasma membrane permeabilization using digitonin (Sigma-Aldrich, Munich, Germany) as reported [20]. Subsequently, cytosolic activity of lysosomal acid phosphatase was assessed by measuring cleavage of a specific substrate using a commercially available assay (Sigma-Aldrich, Munich, Germany).

### Analysis of inflammasome activation

RPE cells were primed with 4 ng/ml human recombinant IL-1α (R&D Systems, Wiesbaden, Germany) for 48 h [7] and macrophages with 200 ng/ml lipopolysaccharide (LPS, from *Escherichia coli* 0127:B8; Sigma-Aldrich, Munich, Germany) for 6 h. For analysis of caspase-1 activity, a fluorochrome-labeled inhibitor of caspase (FLICA) detection assay specific for caspase-1 [carboxyfluorescein-Tyr-Val-Ala-Asp-fluoromethylketone (FAM-YVAD-FMK); Immunochemistry Technologies, Bloomington, MN] was used according to the manufacturer’s instructions. Caspase-1 activation was documented by fluorescence microscopy and quantified by flow cytometry (FACS Canto II; BD Bioscience, Heidelberg, Germany). Interleukin secretion following inflammasome activation in RPE cells was measured by specific ELISAs for human IL-1β (BD Biosciences, Heidelberg, Germany) and human IL-18 (BD Bioscience). Inflammasome activation in murine macrophages was assessed by an ELISA against murine IL-1β (R&D Systems, Wiesbaden, Germany).

### NLRP3 siRNA knockdown

Lipofuscin accumulation was induced in ARPE-19 cells by incubation with HNE-modified POS as described above. Then, cells were transfected with 100 nM small interfering RNA (siRNA) targeting human NLRP3 (Ambion Silencer Select siRNA, ID s41556; Life Technologies, Darmstadt, Germany) or 100 nM nonspecific siRNA (Ambion Silencer Select Negative Control siRNA; Life Technologies) for 24 h using a transfection reagent (Invitrogen Lipofectamin RNAiMax; Life Technologies) according to the manufacturer’s instructions [21]. Subsequently, cells were primed with IL-1α and irradiated with blue light as described above.

### Statistical analysis

Experiments were performed in doublets (Figs. 5 and 6) or triplets (all other experiments). Results are presented as mean ± standard deviation. Statistical analysis was performed using one-tailed (Fig. 7c) or two-tailed (all other experiments) unpaired Student’s *t* tests. Differences were considered statistically significant at *p* < 0.05. In all figures, significance levels as compared to controls are indicated using * for *p* < 0.05, ** for *p* < 0.01, and *** for *p* < 0.001.

## Results

### Blue light irradiation of lipofuscin-loaded RPE cells induces photooxidative damage

**Fig. 1:**
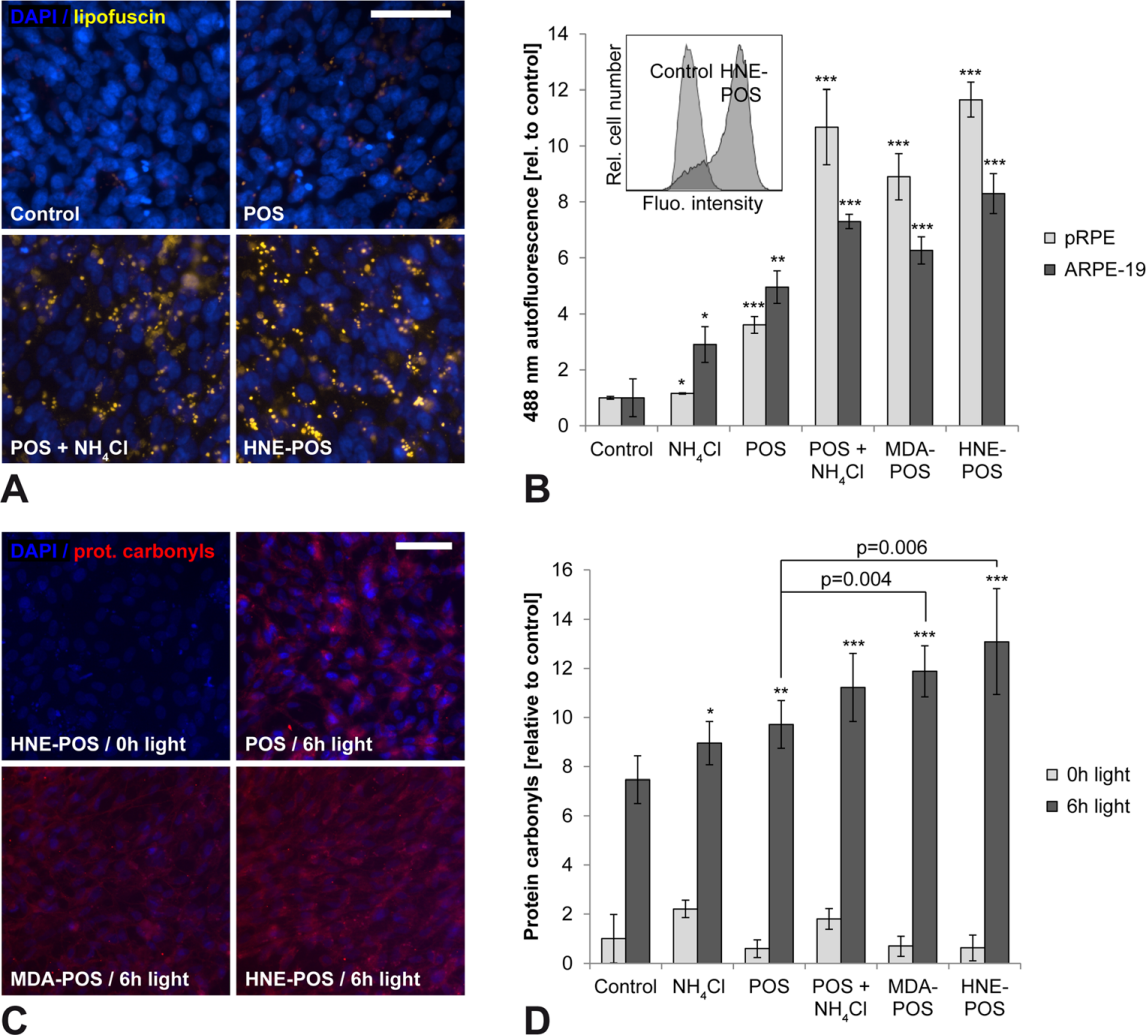
Blue light irradiation of lipofuscin-loaded RPE cells induces photooxidative damage. **a** Intracellular lipofuscin accumulation (*yellow*) was documented in ARPE-19 cells by fluorescence microscopy. Nuclei were visualized by DAPI staining (*blue*). **b** Lipofuscinogenesis was quantified in pRPE cells and ARPE-19 cells by flow cytometry measurements of mean cellular autofluorescence intensity. An example result is shown as an *insert*. Cells were left untreated (*control*) or incubated with the lysosomal inhibitor ammonium chloride (*NH*_*4*_ *Cl*), unmodified photoreceptor outer segments (*POS*), POS and ammonium chloride (*POS + NH*_*4*_ *Cl*), malondialdehyde-modified POS (*MDA-POS*), and 4-hydroxynonenal-modified POS (*HNE-POS*). **c** Photooxidative damage was assessed in ARPE-19 cells by immunocytochemical detection of protein carbonyls (*red*). Nuclei were labeled by staining with DAPI (*blue*). **d** Mean cellular fluorescence of immunocytochemically labeled protein carbonyls was quantified by flow cytometry. *Scale bars*, 50 μm

Lipofuscinogenesis was induced in pRPE cells and ARPE-19 cells either by incubation with isolated photoreceptor outer segments (POS) and simultaneous inhibition of lysosomal degradation by ammonium chloride (NH_4_Cl) or by incubation with POS stabilized against lysosomal degradation by covalent modification with lipid peroxidation products malondialdehyde (MDA) or 4-hydroxynonenal (HNE) as described [16, 18, 22]. Lipofuscinogenesis was evaluated by assessing the accumulation of granular material with lipofuscin-like autofluorescence using fluorescence microscopy and flow cytometry (Fig. 1a,b). Consistent with our previous reports, lipofuscin accumulation was significantly increased in all three treatment groups compared to control groups of cells treated with either native POS alone, with the lysosomal inhibitor alone, or left untreated. The finding that cells treated with the lysosomal inhibitor generate some amount of lipofuscin even in the absence of POS is attributable to impaired autophagy as described before [16]. The spectral profiles of lipofuscin generated in this in vitro model have been reported [16].

Following lipofuscin loading, RPE cells were irradiated with blue light to induce photooxidative stress. Immunocytochemistry confirmed that blue light irradiation resulted in oxidative damage by lipid peroxidation-mediated formation of protein carbonyls (Fig. 1c,d). Even control cells exhibited some photooxidative damage secondary to blue light irradiation. However, photooxidative damage was significantly increased in lipofuscin-loaded cells as compared to controls, consistent with the known photoreactive properties of lipofuscin. Moreover, cells incubated with MDA- or HNE-modified POS exhibited significantly more photooxidative damage compared to cells treated with unmodified POS. We thus verified that the lipofuscin-like material generated by RPE cells in our in vitro model possesses photoreactive properties that result in photooxidative cell damage, similar to lipofuscin generated in human RPE in vivo [14].

### Blue light irradiation of lipofuscin-loaded RPE cells causes phototoxic cell death

**Fig. 2:**
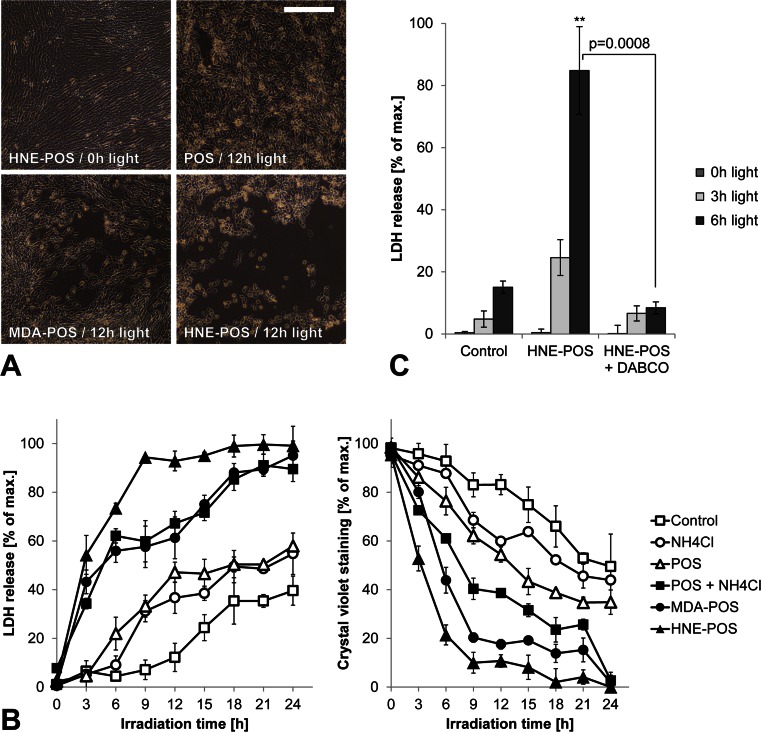
Blue light irradiation of lipofuscin-loaded RPE cells causes phototoxic cell death. **a** Photooxidative damage-induced cytotoxicity was documented in ARPE-19 cells by light microscopy. **b** To quantify the phototoxic effect, both loss of plasma membrane integrity and loss of cellular adhesion were analyzed by measuring LDH release and crystal violet staining, respectively. **c** Photooxidative damage secondary to irradiation was reduced by incubation with the singlet oxygen scavenger DABCO. *Scale bar*, 250 μm

As excessive photooxidative damage may result in cell death, we aimed to determine the phototoxic threshold in our cell culture model by measuring cytotoxicity following increasing durations of irradiation of up to 24 h (Fig. 2a,b). Cytotoxicity was assessed in parallel by means of loss of plasma membrane integrity (LDH release assay) and loss of cell adhesion (crystal violet assay). Consistently in both assays, cytotoxicity increased with blue light dose up to an irradiation time of 24 h when cytotoxicity was nearly complete (90–99 %) in all three lipofuscin-loaded treatment groups. Reduction of photooxidative damage by cell incubation with the singlet oxygen scavenger DABCO during irradiation treatment suppressed the cytotoxic effect (Fig. 2c). These results confirm that in our RPE cell culture model, lipofuscin generation from phagocytosed POS renders the cells susceptible to photooxidative damage. Based on the results, we selected an irradiation duration of 3 and 6 h for our further experiments as for these irradiation times the detected cytotoxic effect of irradiation is limited.

### Lipofuscin-mediated photooxidative damage results in lysosomal membrane permeabilization with cytosolic leakage of lysosomal enzymes

**Fig. 3:**
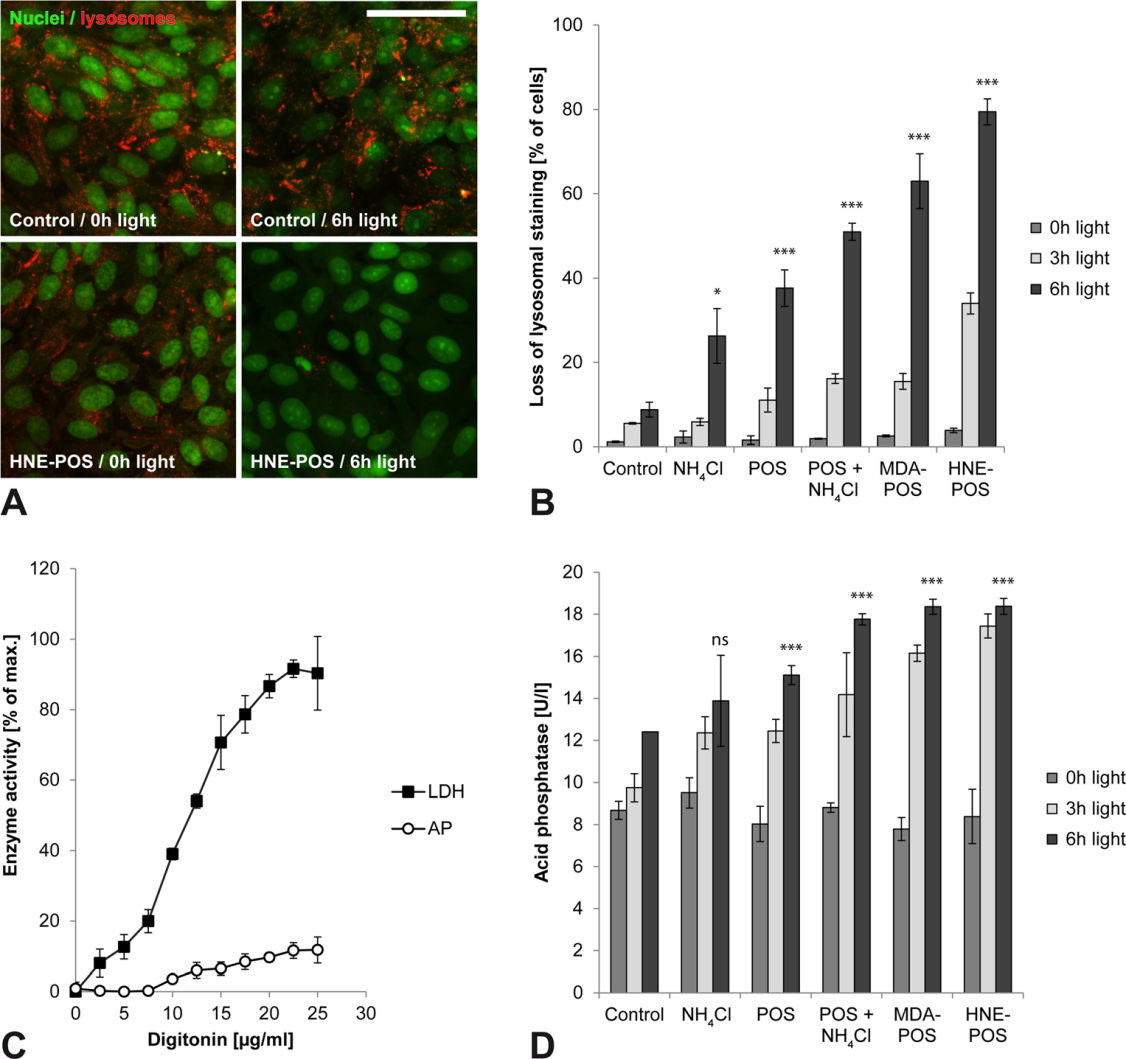
Lipofuscin-mediated photooxidative damage results in lysosomal membrane permeabilization with cytosolic leakage of lysosomal enzymes. **a** Intact lysosomes (*red*) and nuclei (*green*) were visualized in ARPE-19 cells by means on acridine orange staining. **b** Lysosomal membrane permeabilization resulted in a loss of lysosomal staining that was quantified by flow cytometry. **c** Digitonin effect on ARPE-19 cells was titrated for maximum plasma membrane permeabilization (release of cytosolic LDH) and at the same time minimal lysosomal membrane permeabilization (release of lysosomal acid phosphatase, *AP*). **d** A digitonin concentration of 20 μg/ml was selected for separation of cytosolic and lysosomal cellular fractions, and cytosolic leakage of lysosomal enzymes was assessed by analyzing the activity of lysosomal marker enzyme acid phosphatase in the cytosolic fractions. *Scale bar*, 50 μm

Photoreactivity of intralysosomal lipofuscin can induce lysosomal destabilization [14, 15]. We therefore investigated the effects of lipofuscin photoreactivity on lysosomal membrane stability in our model. Lysosomal staining by acridine orange demonstrated intact lysosomes in lipofuscin-loaded cells without irradiation treatment as well as in irradiated cells without lipofuscin loading (Fig. 3a). In contrast, cell treatment with both lipofuscin loading and blue light irradiation resulted in a significant loss of lysosomal staining, indicating lysosomal membrane permeabilization. Quantification of lysosomal staining by flow cytometry demonstrated that loss of lysosomal membrane integrity increased with both light dose and lipofuscin load as measured by means of lipofuscin-like autofluorescence (Fig. 3b).

Cytosolic activity of leaked lysosomal enzymes has been proposed as a mechanism of inflammasome activation [13]. To assess whether lysosomal enzyme leakage occurs in light-exposed RPE cells, we separated cytosolic and lysosomal cellular fractions by plasma membrane permeabilization using digitonin. We employed a digitonin concentration of 20 μg/ml as for this concentration the isolated cytosolic fraction of untreated control cells exhibited a near maximum concentration of the cytosolic marker enzyme LDH while at the same time contamination by the lysosomal marker enzyme acid phosphatase was low (Fig. 3c). Consistent with the results of acridine orange staining, acid phosphatase activity in the isolated cytosolic cell fractions increased with light dose and lipofuscin load (Fig. 3d). The observed background cytosolic acid phosphatase activity that was detectable even in nonirradiated control cells may be explained by partial lysosomal membrane permeabilization by the digitonin treatment as indicated by the digitonin titration curve. Our results demonstrate that lipofuscin-mediated photooxidative damage in RPE cells is associated with lysosomal membrane permeabilization and cytosolic leakage of lysosomal enzymes.

### Lysosomal membrane permeabilization by lipofuscin phototoxicity induces inflammasome activation with activation of caspase-1 and secretion of IL-1β and IL-18

**Fig. 4:**
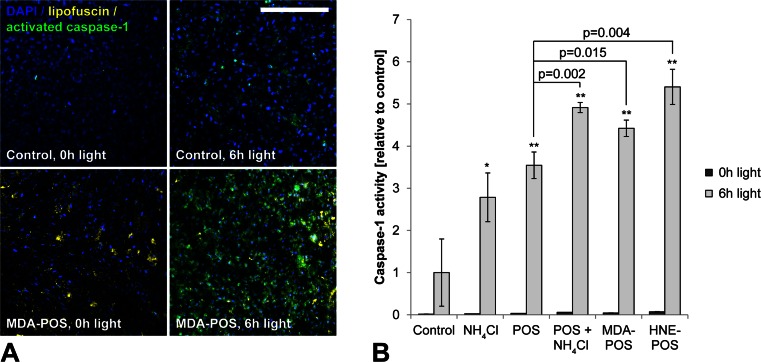
Lysosomal membrane permeabilization by lipofuscin phototoxicity induces activation of caspase-1. **a** Following inflammasome activation, activated caspase-1 was detected in ARPE-19 cells by the FLICA probe FAM-YVAD-FMK (*green*). **b** Caspase-1 activation was quantified by flow cytometry. *Scale bar*, 200 μm

**Fig. 5:**
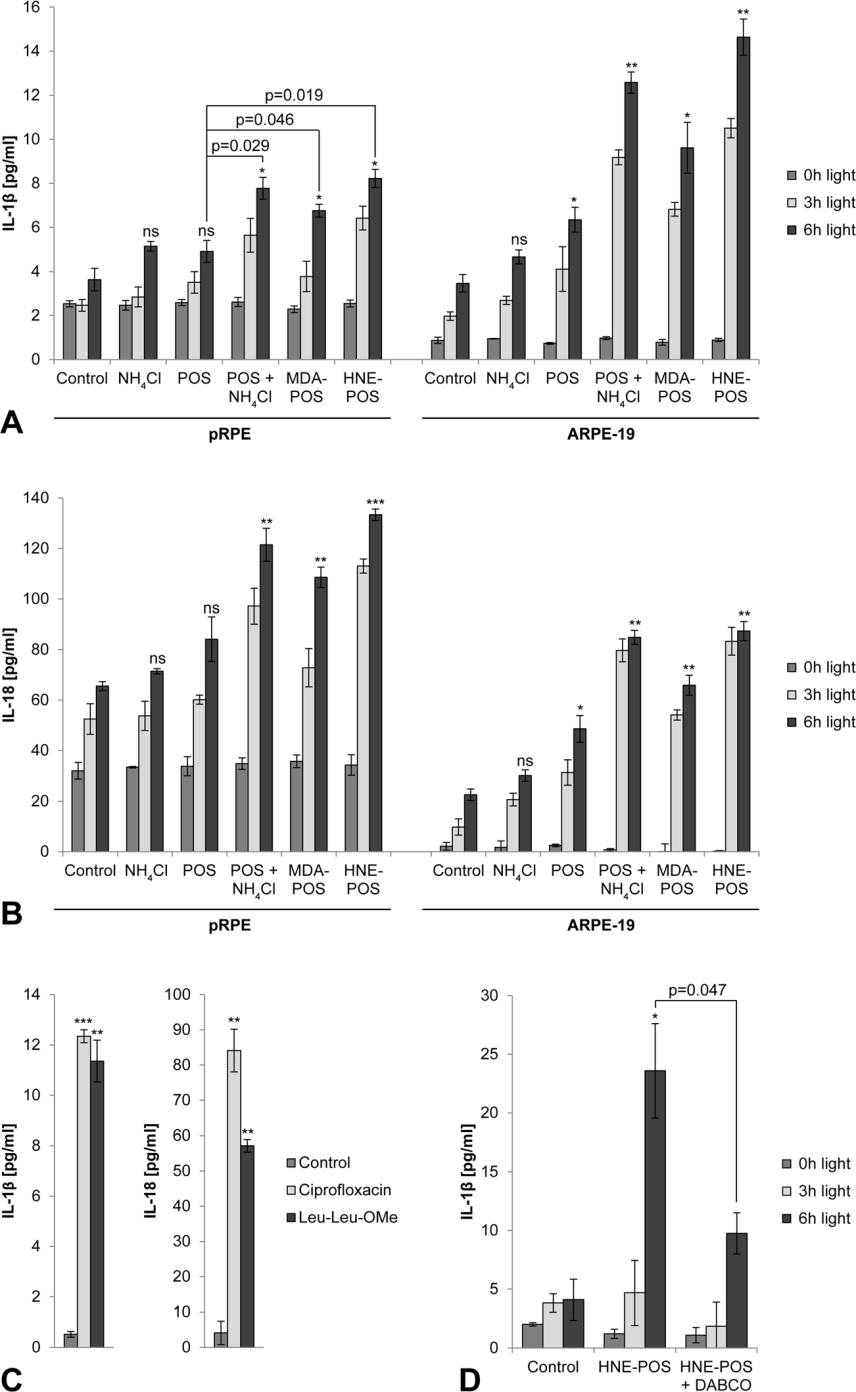
Lysosomal membrane permeabilization by lipofuscin phototoxicity results in inflammasome activation with secretion of IL-1β and IL-18. Inflammasome-mediated secretion of mature IL-1β (**a**) and IL-18 (**b**) was analyzed by ELISA in pRPE cells and ARPE-19 cells. **c** Lysosomal membrane permeabilization by ciprofloxacin and Leu-Leu-OMe in APRE-19 cells served as positive controls. **d** To assess the role of photooxidative damage in irradiation-induced inflammasome activation, ARPE-19 cells were incubation with the singlet oxygen scavenger DABCO during blue light treatment

We then investigated inflammasome activation in RPE cells in response to light treatment. As inflammasome activation requires a priming signal, cells were treated with IL-1α that has been shown to induce NRLP3 inflammasome priming in RPE cells [7]. IL-1α-primed cells were irradiated with blue light, and inflammasome activation was assessed subsequently by measuring caspase-1 activation using the FLICA probe FAM-YVAD-FMK (Fig. 4). We detected significantly increased amounts of activated caspase-1 in lipofuscin-loaded, light-irradiated cells compared to controls. Moreover, secretion of inflammasome-regulated cytokines IL-1β and IL-18 was significantly increased in both pRPE and ARPE-19 cells as measured by ELISA (Fig. 5a,b). Similar to LMP, inflammasome activation increased with light dose and lipofuscin load as measured by autofluorescence. LMP by other means such as treatment with ciprofloxacin [23] or Leu-Leu-OMe [13] likewise induced inflammasome activation in RPE cells (Fig. 5c). Suppression of photooxidative damage by cell incubation with the singlet oxygen scavenger DABCO during irradiation resulted in a significant reduction of irradiation-induced IL-1β secretion (Fig. 5d), thus confirming that photooxidative damage is the critical mechanism underlying inflammasome activation in our experiments.

### Inflammasome activation by lipofuscin phototoxicity is dependent on prior priming, activity of caspase-1, cathepsin B and cathepsin, and expression of NLRP3

**Fig. 6:**
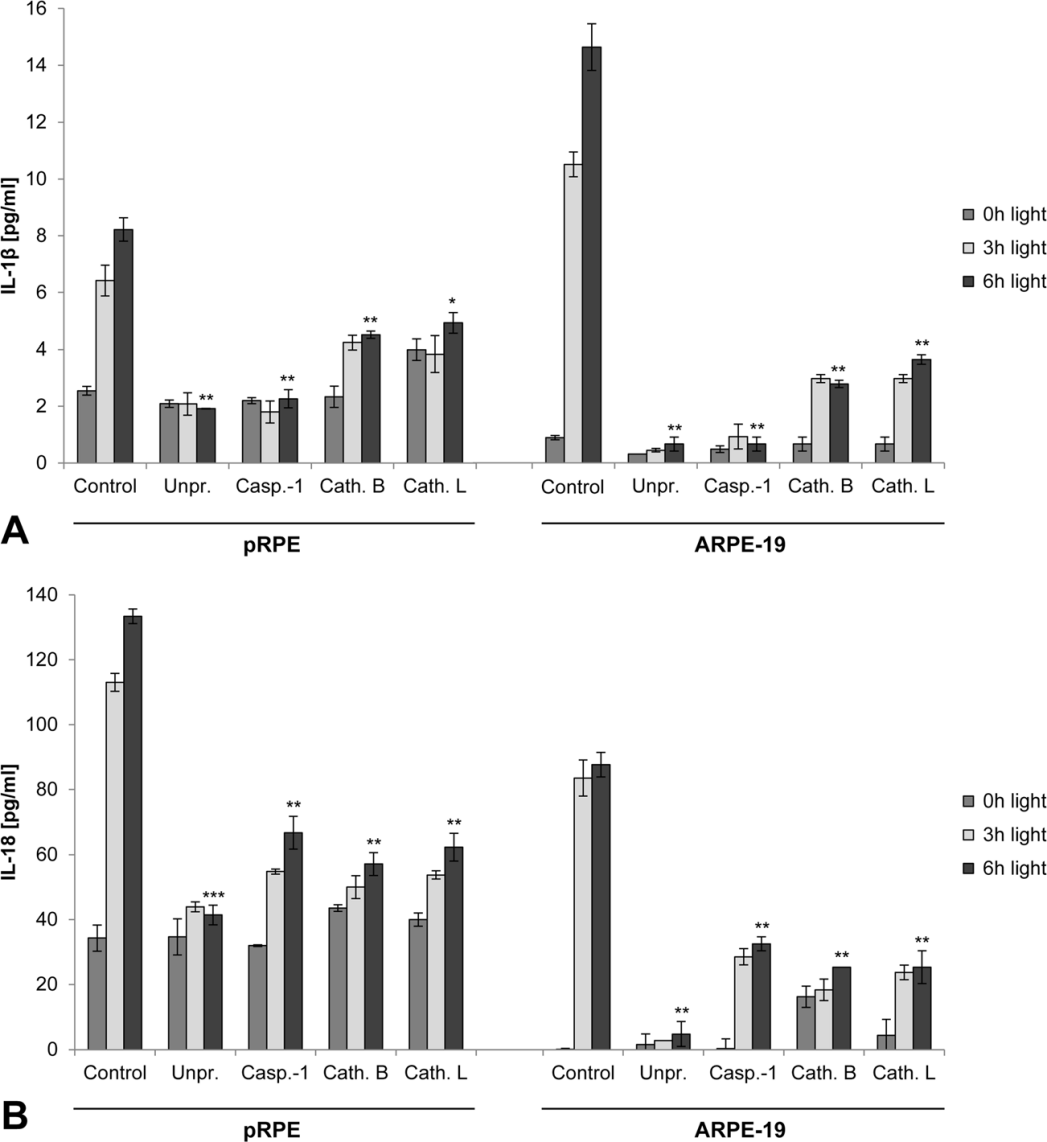
Inflammasome activation by lipofuscin phototoxicity is dependent on inflammasome priming and activity of caspase-1, cathepsin B, and cathepsin L. Secretion of IL-1β (**a**) and IL-18 (**b**) by pRPE cells and ARPE-19 cells was assessed by ELISA in primed cells incubated with HNE-POS (*Control*), unprimed cells incubated with HNE-POS (*Unpr.*), primed cells incubated with HNE-POS and caspase-1 inhibitor Z-YVAD-FMK (*Casp.-1*), primed cells incubated with HNE-POS and cathepsin B inhibitor CA-074 (*Cath. B*), and primed cells incubated with HNE-POS and cathepsin L inhibitor Z-FF-FMK (*Cath. L*)

To further delineate the mechanism by which blue light irradiation induces inflammasome activation in RPE cells, we subjected cells to different inhibitor treatments before and during irradiation (Fig. 6). No inflammasome-mediated secretion of IL-1β and IL-18 was detectable when inflammasome priming by IL-1α prior to irradiation was omitted. Likewise, inhibition of caspase-1 activity by Z-YVAD-FMK suppressed inflammasome activation. Finally, inhibition of the lysosomal proteases cathepsin B or cathepsin L resulted in a significant inhibition of IL-1β and IL-18 secretion, supporting a role of lysosomal enzyme leakage in inflammasome activation in our experiments.

**Fig. 7:**
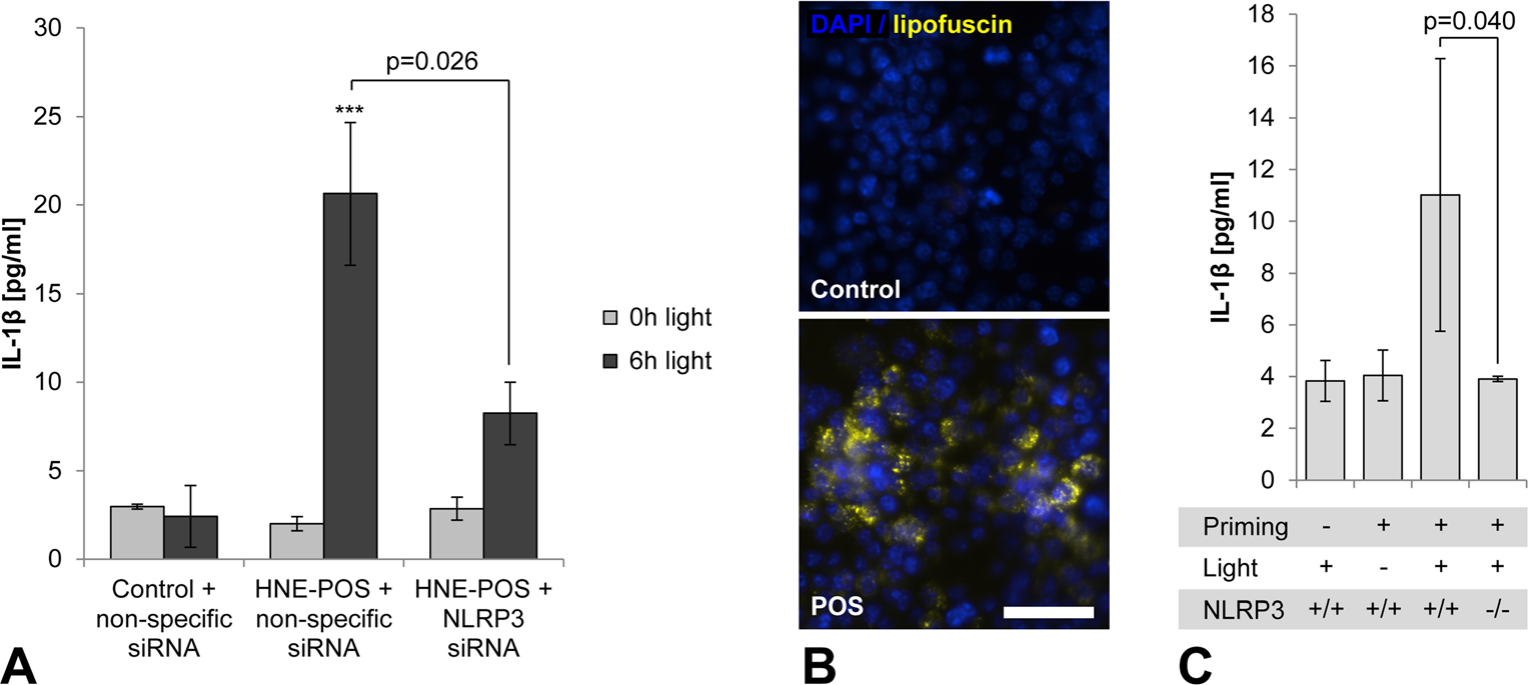
Inflammasome activity by lipofuscin phototoxicity is mediated by NLRP3. **a** In ARPE-19 cells incubated with HNE-POS prior to blue light irradiation, the effect of siRNA-mediated NLRP3 knockdown on IL-1β secretion was assessed as compared to control cells transfected with nonspecific siRNA. **b** Accumulation of lipofuscin-like material (*yellow*) following incubation with POS was documented by fluorescence microscopy in murine macrophages as a substitute model of RPE lipofuscinogenesis. **c** Following POS-induced lipofuscin accumulation, inflammasome priming with LPS, and subsequent blue light irradiation for 4 h, secretion of IL-1β by wild-type (NLRP3 +/+) and NLRP3 knockout (NLRP3 −/−) macrophages was analyzed by ELISA. *Scale bar*, 50 μm

Next, we aimed to investigate whether inflammasome activation by lipofuscin phototoxicity is mediated by the NLRP3 inflammasome as opposed to other inflammasome subtypes. For this, we knocked down NLRP3 expression in lipofuscin-loaded RPE cells by transfection with siRNA against NLRP3 prior to blue light irradiation (Fig. 7a). NLRP3 knockdown resulted in a significant reduction of irradiation-induced IL-1β secretion as compared to control cells transfected with nonspecific siRNA. These results demonstrate that inflammasome activation secondary to lipofuscin phototoxicity in RPE cells is mediated by NLRP3.

To verify this result in a knockout model, we employed a NLRP3-deficient immortalized murine macrophage cell line [13] as NLRP3-deficient RPE cells were not available to us. Macrophages have been shown to generate lipofuscin secondary to POS phagocytosis in vitro similarly to RPE cells [24]. We confirmed lipofuscin accumulation in macrophages secondary to incubation with POS by fluorescence microscopy (Fig. 7b). Lipofuscin-loaded wild-type macrophages responded to blue light irradiation similar to RPE cells, i.e., by inflammasome activation with increased IL-1β secretion that was dependent on prior priming of the cells (Fig. 7c). In NLRP3 knockout cells, however, irradiation-induced IL-1β secretion was significantly suppressed compared to wild-type controls, consistent with the results of NLRP3 knockdown in human RPE cells.

## Discussion

In the yet unresolved pathogenesis of AMD, oxidative damage and chronic immune response have been demonstrated to be centrally involved. However, the connection between these two mechanisms is unclear. Using an RPE cell culture model, we demonstrated for the first time that photooxidative stress by irradiation with blue light activates the NLRP3 inflammasome. This activation is mediated by permeabilization of lysosomal membranes with subsequent cytosolic leakage of lysosomal enzymes. It is amplified by the photosensitizer lipofuscin which accumulates in the RPE in vivo with age and has the highest concentration in the macula. Thus, the molecular mechanism of light-induced inflammasome activation in the RPE links key pathogenic factors of AMD and may provide new targets for therapeutic strategies.

Multiple lines of evidence indicate that lipofuscin accumulation has adverse effects on RPE cell homeostasis and function [25, 26]. Moreover, both in vitro and in vivo studies demonstrated that lipofuscin accumulation increases the susceptibility of the RPE to light-induced cell damage and cell loss [14, 15, 27]. Based on this body of experimental data, a role of light-induced RPE cell damage in AMD pathogenesis has been postulated, and several clinical studies have investigated a possible association of visible light exposure and AMD development. However, results so far have been ambiguous with some studies demonstrating an association while others failed to do so. Among the positive results, the Chesapeake Bay Watermen Study reported a significant association between long-term visible or blue light exposure and geographic atrophy or disciform scarring [28]. In the Beaver Dam Study, sunlight exposure was significantly associated with early AMD [29]. Finally, the European Eye Study found a significant association of blue light exposure and neovascular AMD in patients with low antioxidant levels [30]. While the clinical data regarding the role of light damage in AMD pathogenesis remains controversial, the contribution of oxidative damage has been clearly established in interventional clinical studies such as the age-related eye disease study (AREDS) [2].

In our experiments, we employed a model of blue light-induced photooxidative damage, enhanced by cellular loading with lipofuscin-like material, to study the effects of LMP in RPE cells. Different models for the in vitro study of lipofuscin effects in RPE cells have been described [31]. We and others have used A2E-loaded RPE cells as a model of lipofuscin phototoxicity in the past [15] as A2E is considered the major fluorophore of macular lipofuscin. However, recent data derived from human donor eyes has questioned this role [32]. In this study, we therefore employed a system of endogenous lipofuscin generation from phagocytosed POS in human RPE cells. Cellular lipofuscinogenesis was enhanced by POS modifications with products of lipid peroxidation such as HNE and MDA that result in lysosomal dysfunction by mechanisms described before in detail [16, 18, 22]. The levels of POS protein modifications used in our cell culture were quantified previously [18] and were chosen to correspond to the range of carbonyl modifications detected in human cells in vivo [33].

While this model was designed to closely resemble the in vivo situation, the composition of lipofuscin-like material generated in this model over a period of 7 days is likely to differ from the composition of lipofuscin generated in RPE cells in vivo over a human lifetime. However, the aim of this study was to investigate the consequences of light-induced LMP in RPE cells, and our model of lipofuscin generation was appropriate to achieve LMP by blue light irradiation, similar to what has been reported for RPE cells loaded with lipofuscin granules generated in human RPE in vivo [14]. Hence, potential differences in lipofuscin composition in our model compared to lipofuscin granules from human RPE in vivo do not seem to affect light-induced LMP that was the focus of this study.

In our experiments, the control group of RPE cells exhibited some degree of inflammasome activation following blue light irradiation even in the absence of POS, albeit considerable less compared to the treatment groups incubated with modified POS (Figs. 4 and 5). An explanation for this unexpected finding may be lipofuscin generation as a result of incomplete autophagy of endogenous cellular material in control cells [16], an effect that would be pronounced in cells cultured for several weeks in a confluent state as in our experiments. Indeed, fluorescence microscopy demonstrated low amounts of granules with lipofuscin-like autofluorescence even in control cells (Fig. 1a). Consistent with this explanation, both lipofuscin accumulation and inflammasome activation increased further in cells treated with ammonium chloride in the absence of POS compared to control cells (Fig. 1b and 5), likely as a result of autophagy inhibition by ammonium chloride as previously described [16].Secondary to light-induced LMP, we detected leakage of lysosomal enzymes into the cytosol of RPE cells. Inhibition of lysosomal proteases cathepsin B and L suppressed inflammasome activation associated with light-induced LMP. The finding of cathepsin-dependent NLRP3 inflammasome activation secondary to LMP is consistent with previous studies in silica crystal-challenged macrophages [13]. These results suggest a cytosolic substrate of cathepsin proteolytic activity as a critical component of NLRP3 inflammasome activation. However, the molecular mechanism by which cytosolic activity of cathepsins or other lysosomal enzymes induce inflammasome activation has not yet been resolved.

Isolated HNE was reported to activate the NLRP3 inflammasome in RPE cells [12]. In our experiments, we used covalent modification of POS by HNE to induce lipofuscinogenesis. As POS were thoroughly washed following modification to remove unbound HNE, cells were not exposed to isolated HNE in our experiments. Furthermore, we did not observe inflammasome activation in cells incubated with HNE-modified POS without additional irradiation treatment (Fig. 5). This indicates that inflammasome activation by isolated HNE did not play a role in our study.

Similarly, lipofuscin component A2E alone has been demonstrated to induce NLRP3 inflammasome activation in RPE cells [11]. The amount of A2E within the POS-derived lipofuscin in our experiments is unknown but likely to be small compared to RPE lipofuscin in vivo that accumulates over a lifetime. The low A2E content may explain why we did not see an effect of lipofuscin accumulation alone, without additional irradiation treatment, on inflammasome activation in our experiments (Fig. 5). These results suggest that direct, nonphototoxic effects of A2E did not contribute to inflammasome activation in our study.

Proteins modified by lipid peroxidation products, such as carboxyethylpyrrole (CEP)-modified serum albumin, have been reported to be capable of providing the priming signal for subsequent NLRP3 inflammasome activation [9]. We did not investigate CEP-modified albumin in our study, but we did not observe a priming effect of MDA- or HNE-modified POS proteins in our experiments. Indeed, we found that omission of IL-1α priming prevented inflammasome activation in RPE cells despite incubation of the cells with HNE-POS (Fig. 6). Similar results were obtained for MDA-POS (unpublished data). Thus, HNE- and MDA-modified proteins do not seem to be capable of inducing inflammasome priming in RPE cells.

In this study, we demonstrate that photooxidative damage to human RPE cells, intensified by accumulated lipofuscin, causes lysosomal membrane permeabilization and subsequent activation of the NLRP3 inflammasome by leaking lysosomal enzymes, resulting in secretion of inflammatory cytokines IL-1β and IL-18. These results identify blue light damage as a new mechanism of inflammasome activation and thus contribute to our understanding of light damage to the RPE. Moreover, this mechanism represents a novel molecular link between hallmark features of AMD pathogenesis such as photooxidative damage, innate immune response, and RPE cell dysfunction that may provide new therapeutic targets against this blinding disease.

## Abbreviations

A2E: *N*-retinylidene-*N*-retinyl-ethanolamine
AMD: Age-related macular degeneration
ASC: Apoptosis-associated speck-like protein containing a caspase recruitment domain (CARD)
CEP: Carboxyethylpyrrole
DABCO: 1,4-Diazabicyclooctane
FAM-YVAD-FMK: Carboxyfluorescein-Tyr-Val-Ala-Asp-fluoromethylketone
FLICA: Fluorochrome-labeled inhibitor of caspases
HNE: 4-Hydroxynonenal
IL: Interleukin
Leu-Leu-OMe: L-leucyl-L-leucine methyl ester
LPS: Lipopolysaccharide
MDA: Malondialdehyde
NLRP3: Nucleotide-binding oligomerization domain (NOD)-like receptor (NLR) family, pyrin domain-containing protein 3
POS: Photoreceptor outer segments
RPE: Retinal pigment epithelium
Z-FF-FMK: Z-Phe-Phe-fluoromethylketone
Z-YVAD-FMK: Z-Tyr-Val-Ala-Asp-fluoromethylketone
